# Supplementary material for: Circadian clock disruption promotes the degeneration of dopaminergic neurons in male Drosophila
Source: Nat Commun. 2023 Sep 22;14:5908. doi: 10.1038/s41467-023-41540-y (PMC10516932; doi:10.1038/s41467-023-41540-y)
Supplement: Supplementary file 1 — Supplementary Information [file 41467_2023_41540_MOESM1_ESM.pdf]

# Supplementary Materials for

## Circadian clock disruption promotes the degeneration of dopaminergic neurons in male *Drosophila*

Michaëla Majcin Dorcikova, Lou C. Duret, Emma Pottié, and Emi Nagoshi*

*Corresponding author. Email: Emi.Nagoshi@unige.ch

**This file includes:**

Figures S1 to S7

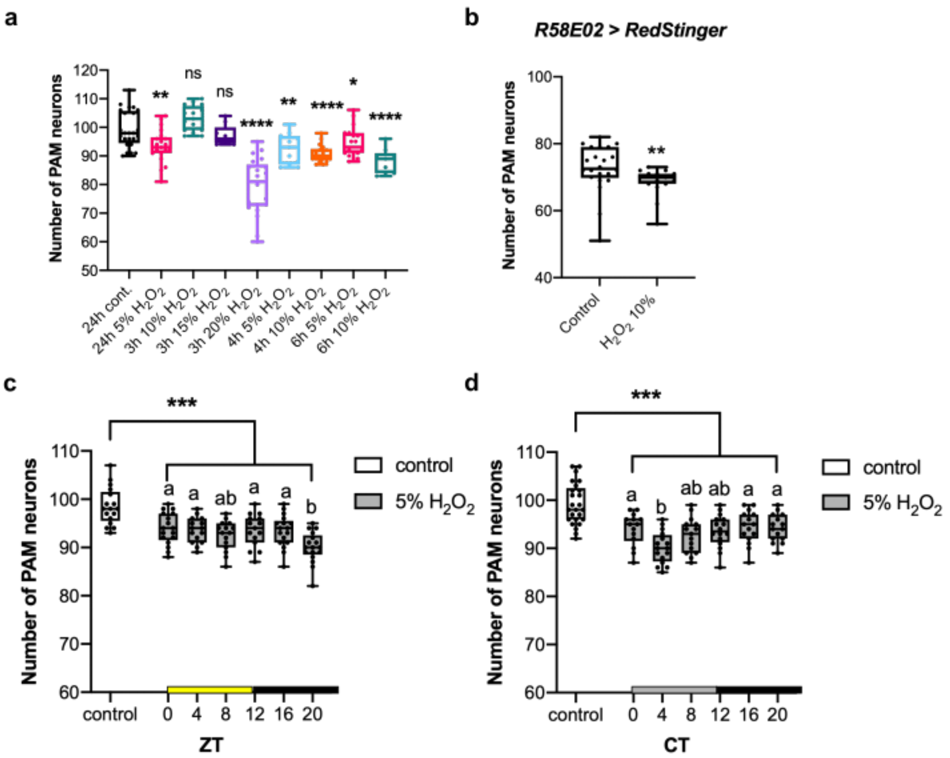


**Figure S1. Short-term $H_2O_2$ treatment induces degeneration of PAM neurons**. (**a**) Effect of percentage and duration of $H_2O_2$ treatment on PAM neurodegeneration. 7-day-old *w*[1118] flies were treated with $H_2O_2$ of indicated doses, starting at ZT1 in LD. PAM neuron counts were examined by anti-TH immunostaining 7 days after the treatment. n = 10–24 hemispheres. *$p$<0.05, **$p$<0.01, and ****$p$<0.0001 by two-tailed t-test comparing with the control treated with water only for 24 h (24 cont.). (**b)** PAM neurons were visualized with *UAS-RedStinger* driven by *R58E02-GAL4* and counted 7 days after a 4-h 10% $H_2O_2$ treatment or control treatment with water only. $H_2O_2$ treatment induces neuronal loss not just a reduction in TH levels. **$p$<0.01 (Mann-Whitney U test). n= 15–23 hemispheres. (**c**, **d)** PAM neuron counts were analyzed by anti-TH immunostaining 7 days after the 4-h 5% $H_2O_2$ treatment performed at different timepoints in LD (**c**) or in DD (**d**). The x-axis indicates the timepoints when $H_2O_2$ was applied. n=14-25 hemispheres. The control group was treated with water only at ZT20 in LD (**c**) and CT20 in DD (**d**). At all timepoints, PAM neuron counts in the $H_2O_2$ treatment group are significantly smaller than those in the control group. ***$p$<0.001 (one-way ANOVA with Tukey's post-hoc test). Within the $H_2O_2$-treated group, flies treated at ZT20 in LD (**c**) and CT4 in DD (**d**) showed a significantly greater cell loss than the treatment at any other timepoint. Different lowercase letters represent statistical significance by ANOVA with Tukey's post-hoc test.

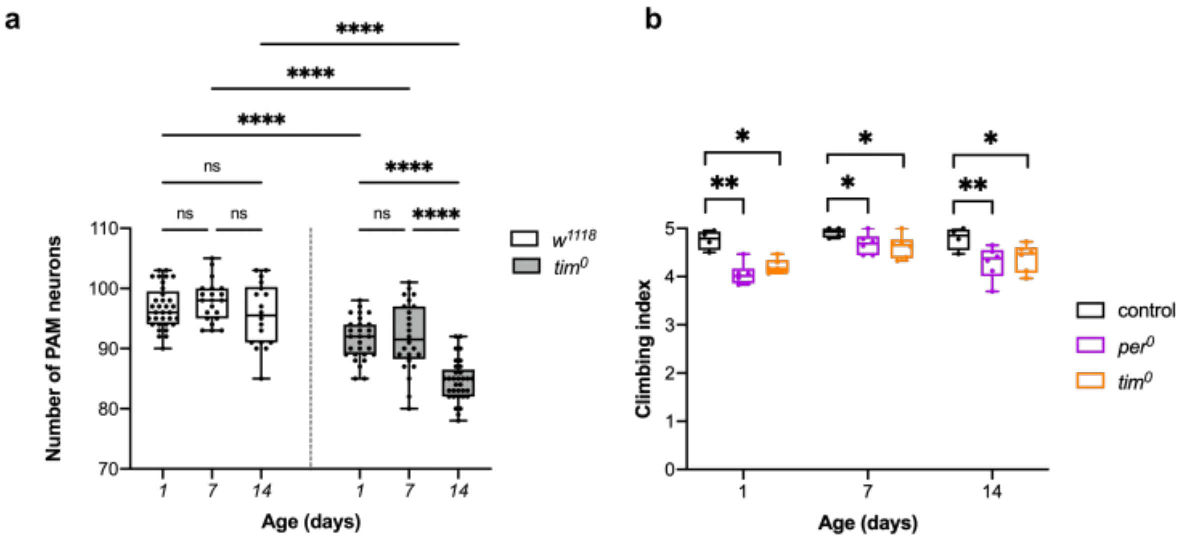


**Figure S2. Progressive loss of PAM neurons in clock gene mutant flies.** (**a**) The number of PAM neurons in *w^1118* and *tim^0* at the indicated ages was analyzed by anti-TH immunostaining. *tim*^0 flies display developmental and age-dependent loss of PAM neurons. n = 18–33 hemispheres. *****p*<0.0001 (one-way ANOVA with Tukey's post-hoc test). (**b**) Negative geotaxis assays performed on *CS*, *per^0*, and *tim^0* flies at the indicated ages show climbing defects in clock gene mutant flies. 4–6 independent experiments. **p*<0.05 and ***p*<0.01 (t-test with Welch correction).

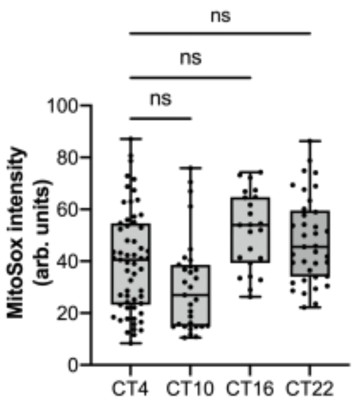


**Figure S3. ROS levels in PAM neurons in DD.** MitoSox red fluorescence levels in PAM neurons were measured at 4 timepoints in DD in 7-day-old *w[1118]* flies. No significant differences were found between CT4 (the timepoint when PAM neurons are most vulnerable to $H_2O_2$) and other timepoints. n = 23–59 flies.

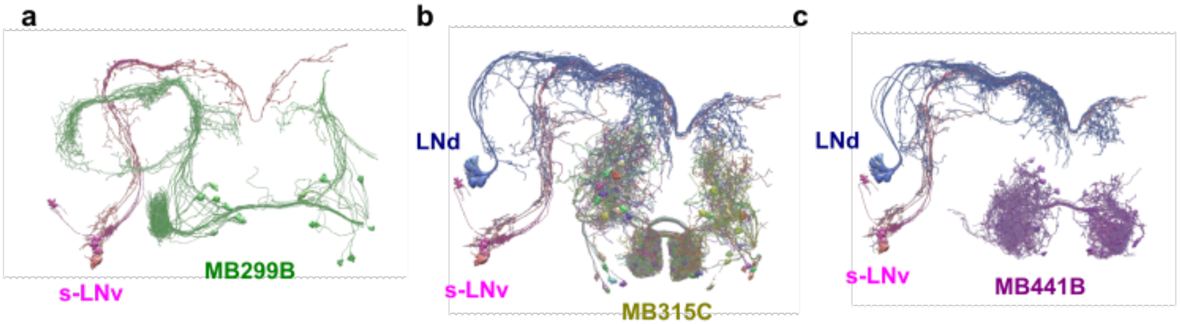


**Figure S4. Position of PAM-$\alpha$1, -$\gamma$5, and -$\gamma$3 neurons and the s-LNv and LNd clock neurons.** (**a**) PAM-$\alpha$1 neurons labeled by the MB299B split-GAL4 driver and the s-LNvs do not contact. (**b**) Projections of PAM-$\gamma$5 neurons expressing MB315C contact the arbors of the LNds. (**c**) Projections of PAM-$\gamma$3 neurons labeled by MB441B do not contact the s-LNvs or the LNds. Images were created using the hemibrain connectome data with the NeuPrint tool.

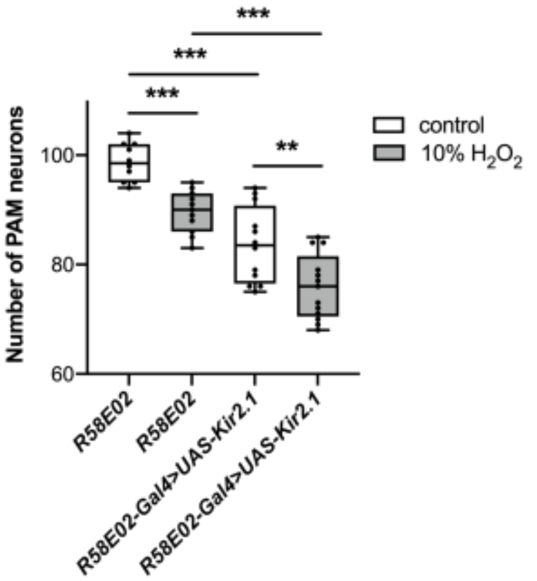


**Figure S5. Effects of electrical silencing on PAM neuron development and degeneration.** Hyperpolarizing Kir2.1 channel was expressed in PAM neurons with the *R58E02* driver. PAM neurons were analyzed by anti-TH immunohistochemistry 7 days after a 4-h 10% $H_2O_2$ treatment performed at ZT20 or the control treatment with water. Kir2.1 expression did not prevent $H_2O_2$-induced PAM neuron loss and reduced the PAM neuron counts in basal conditions. n = 10–13 hemispheres. **$p$<0.01 and ***$p$<0.001 (one-way ANOVA with Tukey's post hoc test).

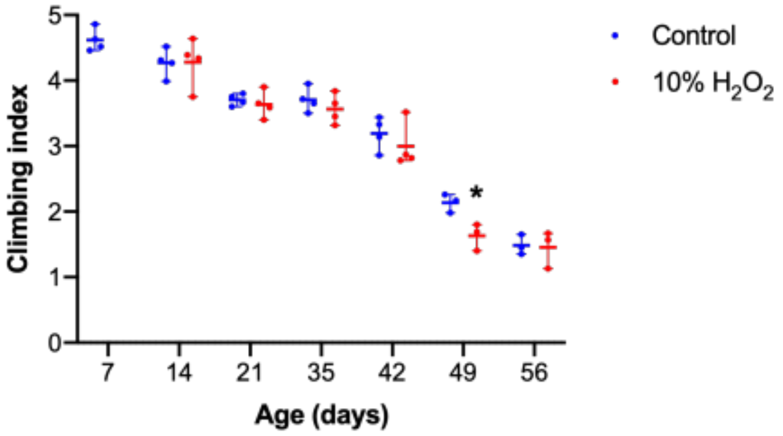


**Figure S6. Short-term $H_2O_2$ treatment does not consistently impair climbing behavior.** The climbing behavior of the *$w^{1118}$* flies was analyzed using the negative geotaxis assay following a 4-h 10% $H_2O_2$ or a control treatment performed at ZT20 at 7 days old; 4 independent experiments. Aside from day 49, no significant differences were observed between the control and the treatment groups throughout aging. *$p$<0.05 (two-tailed t-test).

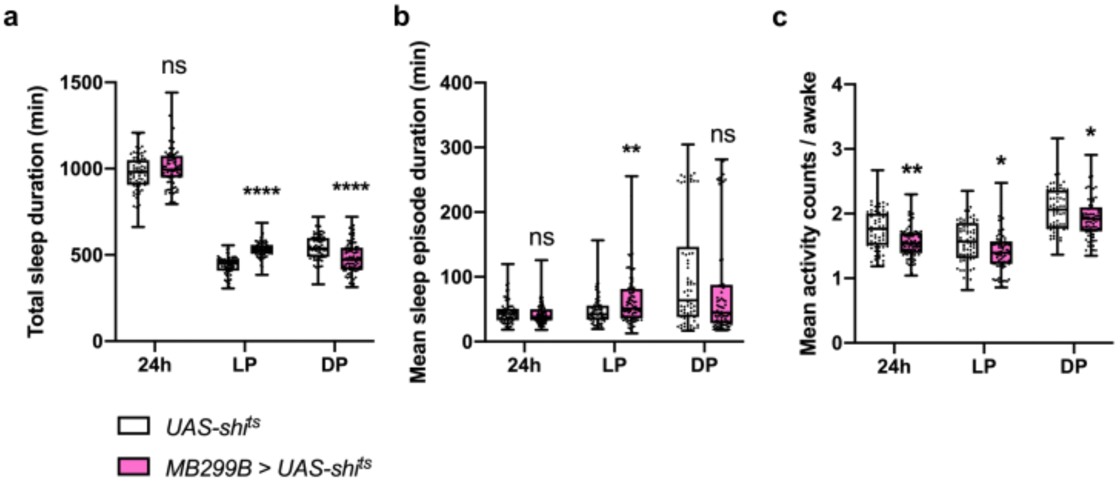


**Figure S7. Blocking output of MB299B neurons modulates sleep and activity levels.** (**a**) MB299B, *UAS-shi*$^{ts}$ flies (MB299B > *UAS-shi*$^{ts}$) and control flies carrying only *UAS-shi*$^{ts}$ were raised at 19°C until 7 days old and then placed in the activity monitor. From the age of 11 days old, the temperature was shifted to 30°C. The sleep and activity of flies from age day 11 to 13 were analyzed and plotted. (**a**) Total sleep duration over 24 h (24h), during daytime (light period, LP) and the night (dark period, DP). (**b**) Mean sleep episode duration. (**c**) Mean activity counts during the wake period. Blocking MB299B neuron output increased daytime sleep but decreased nighttime sleep, whereas activity levels were reduced throughout 24 h by the inhibition of MB299B neuron output. **p*<0.05, ***p*<0.01, and *****p*<0.0001 (two-way ANOVA with Šídák's multiple comparisons test). n = 67–83 flies.
