# Supplementary material for: Circadian clock disruption promotes the degeneration of dopaminergic neurons in male Drosophila
Source: Nat Commun. 2023 Sep 22;14:5908. doi: 10.1038/s41467-023-41540-y (PMC10516932; doi:10.1038/s41467-023-41540-y)
Supplement: Supplementary file 3 — Reporting Summary [file 41467_2023_41540_MOESM3_ESM.pdf]

nature portfolio

Corresponding author(s): Emi Nagoshi

Last updated by author(s): Aug 29, 2023

Reporting Summary

Nature Portfolio wishes to improve the reproducibility of the work that we publish. This form provides structure for consistency and transparency in reporting. For further information on Nature Portfolio policies, see our Editorial Policies and the Editorial Policy Checklist.

Statistics

For all statistical analyses, confirm that the following items are present in the figure legend, table legend, main text, or Methods section.

n/a	Confirmed	
☐	☒	The exact sample size (*n*) for each experimental group/condition, given as a discrete number and unit of measurement
☐	☒	A statement on whether measurements were taken from distinct samples or whether the same sample was measured repeatedly
☐	☒	The statistical test(s) used AND whether they are one- or two-sided<br>*Only common tests should be described solely by name; describe more complex techniques in the Methods section.*
☐	☒	A description of all covariates tested
☐	☒	A description of any assumptions or corrections, such as tests of normality and adjustment for multiple comparisons
☐	☒	A full description of the statistical parameters including central tendency (e.g. means) or other basic estimates (e.g. regression coefficient) AND variation (e.g. standard deviation) or associated estimates of uncertainty (e.g. confidence intervals)
☐	☒	For null hypothesis testing, the test statistic (e.g. *F*, *t*, *r*) with confidence intervals, effect sizes, degrees of freedom and *P* value noted<br>*Give P values as exact values whenever suitable.*
☒	☐	For Bayesian analysis, information on the choice of priors and Markov chain Monte Carlo settings
☒	☐	For hierarchical and complex designs, identification of the appropriate level for tests and full reporting of outcomes
☒	☐	Estimates of effect sizes (e.g. Cohen's *d*, Pearson's *r*), indicating how they were calculated

Our web collection on statistics for biologists contains articles on many of the points above.

Software and code

Policy information about availability of computer code

Data collection	Drosophila Activity Monitor (DAM) Systems 3 (Trikinetics)
Data analysis	GraphPad Prism (v.8.1), NeuPrint

For manuscripts utilizing custom algorithms or software that are central to the research but not yet described in published literature, software must be made available to editors and reviewers. We strongly encourage code deposition in a community repository (e.g. GitHub). See the Nature Portfolio guidelines for submitting code & software for further information.

Data

Policy information about availability of data

All manuscripts must include a data availability statement. This statement should provide the following information, where applicable:

- Accession codes, unique identifiers, or web links for publicly available datasets
- A description of any restrictions on data availability
- For clinical datasets or third party data, please ensure that the statement adheres to our policy

All data are available in the main text or the supplementary materials. Source Data are provided with this paper.

Human research participants

Policy information about studies involving human research participants and Sex and Gender in Research.

Reporting on sex and gender	n.a.
Population characteristics	n.a.
Recruitment	n.a.
Ethics oversight	n.a.

Note that full information on the approval of the study protocol must also be provided in the manuscript.

Field-specific reporting

Please select the one below that is the best fit for your research. If you are not sure, read the appropriate sections before making your selection.

☒ Life sciences ☐ Behavioural & social sciences ☐ Ecological, evolutionary & environmental sciences

For a reference copy of the document with all sections, see nature.com/documents/nr-reporting-summary-flat.pdf

Life sciences study design

All studies must disclose on these points even when the disclosure is negative.

Sample size	Sample sizes were chosen without calculation but based on the literature describing similar experiments. Sample sizes for sleep experiments are similar to those in (Parisky et al. Curr Bio 2016, Guo et al. Nature 2016). Sample sizes for the immunostaining, were ≥ 20 brains per group, which corresponds to the range of the sample sizes used for similar experiments in the literature, such as (Chouhan et al. Nature 2020, Bai et al. Cell Reports 2018).
Data exclusions	Data were excluded from the analysis only when the animal's viability was severely impaired. (Dead flies during the calcium imaging.)
Replication	All the experiment were repeated at least twice. All attempts at replication were successful.
Randomization	Experiments were not randomized, since all the experiments included one or more a control group(s) and only one variant was analyzed at a time.
Blinding	Investigators were not blinded to genotypes and conditions in most experiments, because only one experimenter has conducted the entire course of the experiment. However, all data were analyzed using unbiased statistical methods. Experiments performed by multiple investigators (Fig. 2a-d, Fig.S2a-b, Fig.3d-e, Fig. 4c, and Fig.S7a-c) were analyzed blindly to the genotype and conditions.

Reporting for specific materials, systems and methods

We require information from authors about some types of materials, experimental systems and methods used in many studies. Here, indicate whether each material, system or method listed is relevant to your study. If you are not sure if a list item applies to your research, read the appropriate section before selecting a response.

Materials & experimental systems

n/a	Involved in the study	
☐	☒	Antibodies
☒	☐	Eukaryotic cell lines
☒	☐	Palaeontology and archaeology
☐	☒	Animals and other organisms
☒	☐	Clinical data
☒	☐	Dual use research of concern

Methods

n/a	Involved in the study	
☒	☐	ChIP-seq
☒	☐	Flow cytometry
☒	☐	MRI-based neuroimaging

Antibodies

Antibodies used	rabbit polyclonal anti-TH (Millipore, ab152), mouse monoclonal antibody nc82 (Developmental Studies Hybridoma Bank), polyclonal rabbit anti-GFP (Invitrogen, A6455), monoclonal mouse anti-HA (Covance, MMS-101P), Alexa Fluor anti-rabbit 488 (Thermofisher, A21052) , Alexa Fluor anti-mouse 633 (Thermofisher, A11008)

Validation	The antibodies from commercial sources and have been validated by the vendors and also in our previous studies (Miozzo et al. 2022; Machado Almeida et al. 2021).

Animals and other research organisms

Policy information about studies involving animals; ARRIVE guidelines recommended for reporting animal research, and Sex and Gender in Research

Laboratory animals	Drosophila melanogaster (w1118, CS). Age between 1- to 80-day-old.
Wild animals	This study did not use wild animals
Reporting on sex	We did not observe any difference between males and females in immunohistochemisty and imaging based analysis during the pilot experiments. Therefore, male flies were used in this study.
Field-collected samples	This study did not use field-collected samples.
Ethics oversight	This study does not involve vertebrate animals and does not require ethics oversight.

Note that full information on the approval of the study protocol must also be provided in the manuscript.
